# High serum proteinase-3 levels predict poor progression-free survival and lower efficacy of bevacizumab in metastatic colorectal cancer

**DOI:** 10.1186/s12885-024-11924-4

**Published:** 2024-02-02

**Authors:** Kei Furuya, Masao Nakajima, Ryouichi Tsunedomi, Yuki Nakagami, Ming Xu, Hiroto Matsui, Yukio Tokumitsu, Yoshitaro Shindo, Yusaku Watanabe, Shinobu Tomochika, Noriko Maeda, Michihisa Iida, Nobuaki Suzuki, Shigeru Takeda, Shoichi Hazama, Tatsuya Ioka, Yoshinobu Hoshii, Tomio Ueno, Hiroaki Nagano

**Affiliations:** 1 Department of Gastroenterological, Breast and Endocrine Surgery, Yamaguchi University Graduate School of Medicine, 1-1-1 Minami-Kogushi, Ube, Yamaguchi 755-8505 Japan; 2 Oncology Center, Yamaguchi University Hospital, Ube, Yamaguchi 755-8505 Japan; 3 Department of Diagnostic Pathology, Yamaguchi University Hospital, Ube, Yamaguchi 755-8505 Japan; 4 Department of Digestive Surgery, Kawasaki Medical School, Kurashiki, Okayama 701-0192 Japan

**Keywords:** Biomarker, Bevacizumab, Chemotherapy, Metastatic colorectal cancer, Proteinase-3

## Abstract

**Background:**

To improve the prognosis of patients with metastatic colorectal cancer (mCRC), investigating predictive biomarkers of their prognosis and chemotherapeutic responsiveness is necessary. This study aimed to analyze the clinical significance of serum proteinase-3 (PRTN3) as a predictor for prognosis and chemosensitivity, especially to bevacizumab therapy, in mCRC.

**Methods:**

This single-center retrospective observational study enrolled 79 patients with mCRC in our hospital and 353 patients with colorectal cancer in the TCGA database. Preoperative serum PRTN3 levels were measured using an enzyme-linked immunosorbent assay. The clinicopathological characteristics and prognosis according to serum PRTN3 levels were then evaluated. PRTN3 expression in tumor and stromal cells was evaluated immunohistochemically. The impact of PRTN3 levels on angiogenesis and bevacizumab sensitivity was evaluated using the tube formation assay.

**Results:**

Serum PRTN3 levels were an independent poor prognostic factor for progression-free survival (PFS) (hazard ratio, 2.082; 95% confidence interval, 1.118-3.647; *P*=0.010) in patients with mCRC. Similarly, prognostic analysis with TCGA data sets showed poorer overall survival in patients with PRTN3 expression than that in patients without PRTN3 expression, especially in patients with stage IV. Immunohistochemical analysis of resected specimens revealed that stromal neutrophils expressed PRTN3, and their expression level was significantly correlated with serum PRTN3 levels. Interestingly, the effectiveness of first-line chemotherapy was significantly poorer in the high serum PRTN3 level group. High serum PRTN3 was significantly associated with poor PFS (hazard ratio, 3.027; 95% confidence interval, 1.175–7.793; *P*=0.0161) in patients treated with bevacizumab, an anti-angiogenic inhibitor. The tube formation assay revealed that PRTN3 administration notably augmented angiogenesis while simultaneously attenuating the anti-angiogenic influence exerted by bevacizumab therapy.

**Conclusions:**

Serum PRTN3 levels could be a novel predictive biomarker of PFS of first-line chemotherapy, especially for bevacizumab therapy, in patients with mCRC

**Supplementary Information:**

The online version contains supplementary material available at 10.1186/s12885-024-11924-4.

## Background

Colorectal cancer (CRC) is the third most common cancer worldwide, accounting for approximately 10% of all cancer cases and is the second leading cause of cancer-related deaths worldwide. The projected statistics for CRC by the year 2040 indicate a significant upward trend in its incidence and mortality rates. Its risk factors include an unhealthy lifestyle, such as obesity, smoking, and excessive alcohol consumption, which requires timely diagnosis, appropriate treatment, and regular follow-up to improve survival rates [1]. Treatments for CRC are based on the type and progression of the cancer. The standard treatment for metastatic CRC (mCRC) is a multidisciplinary approach that involves chemotherapy, immunotherapy, and surgery [2–4]. Moreover, the therapeutic efficacy of chemotherapy needs to be enhanced to improve mCRC survival. The additive or synergistic effects of the combination of anti-vascular endothelial growth factor (VEGF) monoclonal antibodies (mAbs), such as bevacizumab (Bev), and cytotoxic agents have been shown to overcome resistance to chemotherapy [5]. The first hypothesis for this mechanism is the direct anti-vascular effects of cytotoxic agents that intensify the pro-apoptotic effects of anti-VEGF mAbs on the vascular endothelium [6]. Another hypothesis is that the normalization of tumor vessels by anti-VEGF mAbs increases the uptake of cytotoxic agents and antibodies [5, 7]. Anti-VEGF mAbs, especially Bev, are currently the standard of care for mCRC [2]. Using Bev with chemotherapy has a significantly higher PFS than that resulting from using placebo with chemotherapy (the median PFS: 9.4 months versus 8.0 months), and the response rate was approximately 40% [8]. The overall incidence of predefined grade 3/4 adverse events related to bevacizumab therapy is 16%. These adverse events included hypertension, proteinuria, gastrointestinal perforation, and stroke [8]. Given that some patients do not benefit from Bev therapy, another approach is to optimize the benefits of chemotherapy regimens using biomarkers that predict chemosensitivity. However, only few biomarkers (e.g., RAS mutations status, and UGT1A1 polymorphism) have been identified to guide chemotherapy selection in clinical practice [2, 9]. Moreover, some of these markers require invasive measurements. Furthermore, no clinical and biological factors relating to Bev therapy have been identified, despite the extensive research done to explore the predictive biomarkers of response to Bev therapy [10]. Therefore, more predictive but less invasive biomarkers than those currently available need to be explored [11–14].

We have previously explored prognostic biomarkers of CRC by comprehensive proteomics analysis [15–17]. Proteinase-3 (PRTN3) belongs to the neutrophil-derived and serine protease families and is stored within azurophil granules [18]. PRTN3 is involved in neutrophil differentiation and proliferation, inflammation, and vasculitis [19, 20], as well as in the invasion of tumor cells and endothelial cells through the activation of matrix metalloproteinases [21, 22]. Furthermore, PRTN3 expression in tumor tissue is associated with poor prognosis in some carcinomas [23–25]. However, the relationship between serum PRTN3 levels and colorectal cancer is still unclear. Thus, this study aimed to analyze the clinical significance of serum PRTN3 as a predictor of chemosensitivity and prognosis in mCRC.

## Methods

### Study design and patients

**Fig. 1:**
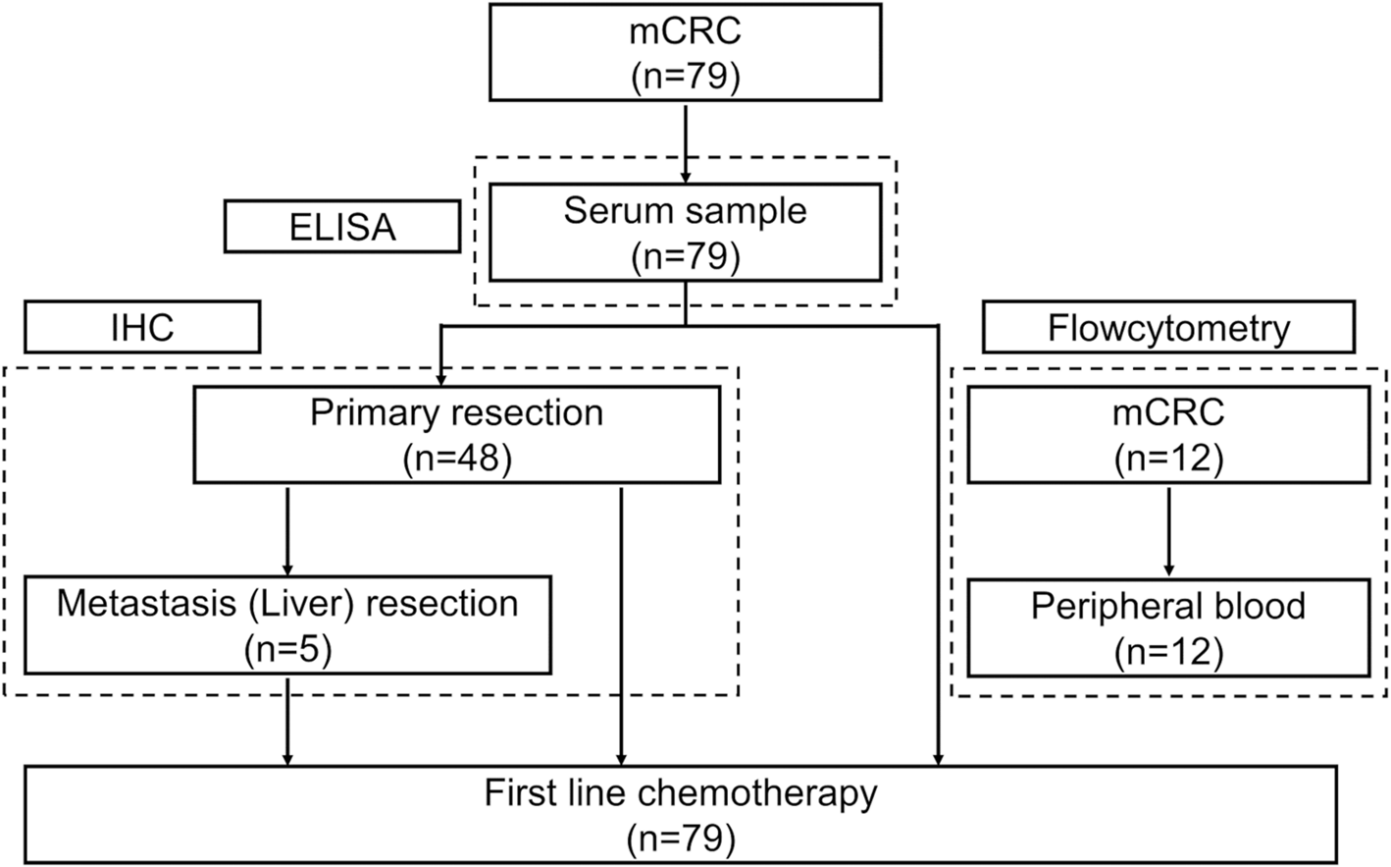
Consort diagram of this study. In total, 79 patients with mCRC are enrolled in the study. Patient sera are used to measure PRTN3 levels with ELISA. Overall, 48 primary tumor and 5 liver tumor samples obtained surgically from the 79 patients are subjected to IHC staining. The prognostic impact and association with the efficacy of first-line chemotherapy of PRTN3 levels are examined. Flow cytometric analysis is performed using peripheral whole blood samples from 12 patients with mCRC. Abbreviations: IHC, immunohistochemistry; mCRC, metastatic colorectal cancer; PRTN3, proteinase-3

This was a single-center, retrospective, observational study. From January 2008 to December 2019, 79 serum samples were obtained from consecutive patients with mCRC at our hospital and stored at -80 °C until use. Of the 48 patients who underwent surgery before chemotherapy, 48 primary tumors and 5 metastatic liver tumors were surgically obtained. Peripheral whole blood samples for flow cytometric analysis were obtained from 12 patients with mCRC (Fig. 1). Computed tomography was performed every 2-3 months to evaluate treatment efficacy. Chemotherapy efficacy was evaluated by examining the remaining target lesions in patients who did not undergo radical resection, otherwise the efficacy was evaluated during preoperative chemotherapy in patients with radical resection according to RECIST criteria version 1.1.

This study was approved by the Ethics Committee of Yamaguchi University Hospital (H20-102, H23-135, and H28-074) and was performed in accordance with the Declaration of Helsinki.

### Comprehensive proteomics analysis of serum and tumor tissue

Comprehensive analysis of protein levels in the pretreatment serum and tumor tissue lysates of 24 patients with mCRC was performed using SOMAscan (Soma Logic, Boulder, CO) following the manufacturer’s protocol (Soma Logic) [15, 16]. Briefly, total protein in frozen CRC samples was quantified by adding a Halt Protease Inhibitor Cocktail (Thermo Fisher Scientific, Kanagawa, Japan) using a Qiagen Tissue Lyser (Qiagen Scientific, Tokyo, Japan). Samples were sent to Soma Logic and analyzed using the SOMAscan assay. SOMAscan readings are displayed as relative fluorescence units.

### Analysis of the relationship between PRTN3 gene expression and mCRC prognosis using The Cancer Genome Atlas database

Transcriptional and corresponding clinical information of 353 CRC patients was obtained from The Cancer Genome Atlas [26]. Survival curve analysis was performed for the PRTN3-expression group (those with mRNA expression quantification of >0 fragments per kilobase of exon per million mapped reads [FPKM]) and the non-expression group (those with mRNA expression quantification of 0 FPKM).

### Measurement of serum PRTN3 levels

Serum PRTN3 concentration was measured using the human PRTN3 enzyme-linked immunosorbent assay (ELISA) kit (ab226902; Abcam, Tokyo, Japan) according to the manufacturer’s instructions. Briefly, after adding 50 µl of a standard or sample solution to appropriate wells, 50 µl of the antibody cocktail (capture antibody and detector antibody) was added to all wells, and the samples were incubated at room temperature for 1 h. After washing, 100 µl of TMB Development Solution was added in each well and incubated for 10 min. The absorbance was read at a wavelength of 450 nm after adding 100 µl of a stop solution. Absorbance was measured using an EnVision Multilabel Plate Reader (PerkinElmer, Waltham, MA, USA). A standard curve was prepared for stock standards. The sensitivity of this assay was 150 pg/ml. All standards, controls, and samples were measured in duplicates.

### Immunohistochemical staining

Immunohistochemistry (IHC) was performed using 4-µm-thick formalin-fixed paraffin-embedded (FFPE) sections. Sections were deparaffinized with xylene and alcohol, and then antibody activation was performed using a 10 mM sodium citrate buffer (Agilent Technologies, Tokyo, Japan) at pH=6.0 for 20 min at 95°C. Endogenous peroxidase activity was blocked with a blocking solution (S2023; Agilent Technologies) for 5 min, followed by blocking non-specific reactions for 10 min at room temperature (24°C) (X0909; Agilent Technologies) and incubating with rabbit anti-human PRTN3 antibody (EPR6277, Abcam; dilution 1:200) at 4°C overnight.

After washing, the sections were incubated with an appropriate secondary antibody (K4003; Agilent Technologies; undiluted) for 30 min at room temperature (24°C). DAB (Agilent Technologies) was used according to the manufacturer’s protocol and allowed to react with the sections for 3 min. Contrast staining was performed with hematoxylin, and the samples were sealed after dehydration. Images were obtained using an all-in-one fluorescence microscope (BZ-X710, KEYENCE, Osaka, Japan). PRTN3 expression in tissue samples was evaluated by a pathology specialist and an investigator who were blinded to the patient outcomes. Focusing on the tumor cells, a semi-quantitative evaluation of PRTN3 expression was performed using the composite expression score (CES) as described previously [27].

CES=4 × (intensity score − 1) + frequency score. Intensity scores: 0 (negative), 1 (weakly positive), 2 (moderately positive), and 3 (strongly positive). The frequency scores were as follows: 1 (5–24%), 2 (25–49%), 3 (50–74%), and 4 (75–100%).

For the analysis of PRTN3 expression in stromal cells, five areas on each slide that were most abundant in positive cells were photographed under ×400 high-power fields, and the number of positive stromal cells was counted.

### Immunofluorescence

Immunofluorescence was performed with 4-µm-thick FFPE sections. After deparaffinization and antigen activation (pH 6.0, 95°C, 20 min), non-specific reactions were blocked. The sections were incubated with the anti-human PRTN3 antibody (at the same dilution rate as that for IHC), anti-CD3 antibody (17A2; Thermo Fisher Scientific, dilution 1:100), anti-CD68 antibody (PG-M1, Abcam; dilution 1:400), and anti-CD66b antibody (G10F5, Novus Biologicals, Centennial, CO; dilution 1:200) at 4°C overnight. The sections were then incubated with secondary antibody mixtures (anti-mouse Alexa Fluor 488; Thermo Fisher Scientific; dilution 1:1000 and anti-rabbit Alexa Fluor 555; Thermo Fisher Scientific; dilution 1:1000) for 40 min at room temperature. Thereafter, they were stained with DAPI for nuclear contrast staining. Images were obtained using an all-in-one fluorescence microscope (BZ-X710, KEYENCE).

### Flow cytometry

Fresh peripheral blood (100 µl) was collected in EDTA-anticoagulation tubes and reacted at room temperature for 30 min in the dark with an anti-PRTN3 antibody (PR3G-2, Abcam; undiluted) and anti-CD66b antibody (G10F5, BioLegend, San Diego, CA; undiluted) according to the manufacturer’s protocol. Erythrocytes were lysed in a lysis solution (BD Biosciences, San Jose, CA, USA) at room temperature (24°C)for 10 min in the dark and centrifuged at 1500 rpm for 10 min. The cells were then washed with PBS. Finally, the cells were resuspended in 500 µl cell FIX (BD Biosciences; dilution, 1:10). Flow cytometric data were acquired using a NovoCyte Flow Cytometer (ACEA Biosciences, San Diego, CA) with the NovoExpress software (version 1.3.0, ACEA Biosciences) and analyzed using the FlowJo software (Tree Star, Ashland, OR, USA).

### Tube formation assay

The tube formation assay was used to study the achieved angiogenesis and to evaluate the effects of specific cell lines and factors such as VEGF on angiogenesis [28]. It was performed using the endothelial tube formation kit (Cell Biolabs, San Diego, USA) according to the manufacturer’s protocol. Briefly, pre-chilled 96-well plates were coated with ECM gel (50 μL/well) and incubated at 37℃ for 1 h. We then added 2.5×10^4^ cells of human umbilical vein endothelial cells (Takara, Tokyo, Japan) and 150 μL culture medium per well. Addition of a combination of PRTN3 and Bev was performed to evaluate the angiogenic capacity of PRTN3 and to confirm whether PRTN3 can overcome the anti-angiogenic effects of Bev therapy. PRTN3 concentrations were set in 10x intervals to include the serum cutoff values (21.6 ng/ml) and only one Bev concentration was set. Briefly, PRTN3 (RPB434Hu02, Cloud Clone Corp., USA) was added at a final concentration of 0, 1, 10, 100, 1000 ng/mL, and Bev (Chugai Pharmaceutical Co., Tokyo, Japan) was added at a final concentration of 0 or 2.5 mg/mL to each well. After incubation at 37℃ and 5% CO^2^ for 2 h, the number of branches and tube area were evaluated using an all-in-one fluorescence microscope (BZ-X710, KEYENCE).

### Statistical analysis

The cutoff value of serum PRTN3 was determined using time-dependent receiver operating characteristic (ROC) curve analysis. The Mann–Whitney U-test was used to examine between-group differences, such as, the CES, the number of PRTN3 positive in stroma relative to its serum levels according to IHC, the percentage of CD66b positive cells relative to that of PRTN positive cells according to Flow cytometry, and the amount of branches and tube area relative to the presence of Bev in Tube formation assay. Differences of tube formation among the groups were estimated using the Kruskal–Wallis test, followed by the Steel–Dwass test. Categorical variables were compared using the chi-squared test. Overall survival (OS) was defined from the date of initial diagnosis to the date of death. Survival curves were generated using the Kaplan–Meier method and compared using the log-rank test. Cox’s proportional hazards model was used to estimate the hazard ratio (HR) and perform univariate and multivariate analyses. The variables included in the univariate analysis for assessing prognosis were as follows: PRTN3 levels, age, participants’ sex, WBC levels, neutrophil count, tumor markers levels, TNM stages, and the status of radical resection. Additionally, variables selected for the multivariate analysis were those with a univariate analysis p-value of < 0.10. Progression-free survival (PFS) for first-line chemotherapy was defined as the interval from the date of the first chemotherapy to the date of progression or death. All statistical analyses were performed using JMP Pro16 (SAS Institute, Cary, NC, USA). Statistical significance was set at *P*<0.05.

## Results

### Identification of PRTN3 as a Predictive Marker for mCRC Prognosis

The good (OS ≥3 years) and poor (OS <2 years) prognosis groups involved 9 and 11 patients, respectively. The candidate proteins ranked according to their Fisher ratio are shown in Supplementary Table 1. Myeloperoxidase was the top prognostic candidate protein in serum, but it had already been reported to be associated with prognosis in CRC [29]. It estimated to be difficult to find its novel roles for mCRC and was thus excluded. The expression of PRTN3, the second candidate protein in serum, was significantly higher in the poor prognosis group than that in the good prognosis group (*P*<0.001). The other candidate proteins were known prognostic markers in patients with mCRC and were therefore also excluded from the analysis [30–32]. In addition, PRTN3 was also one of the top 10 proteins in the tissue samples.

**Fig. 2:**
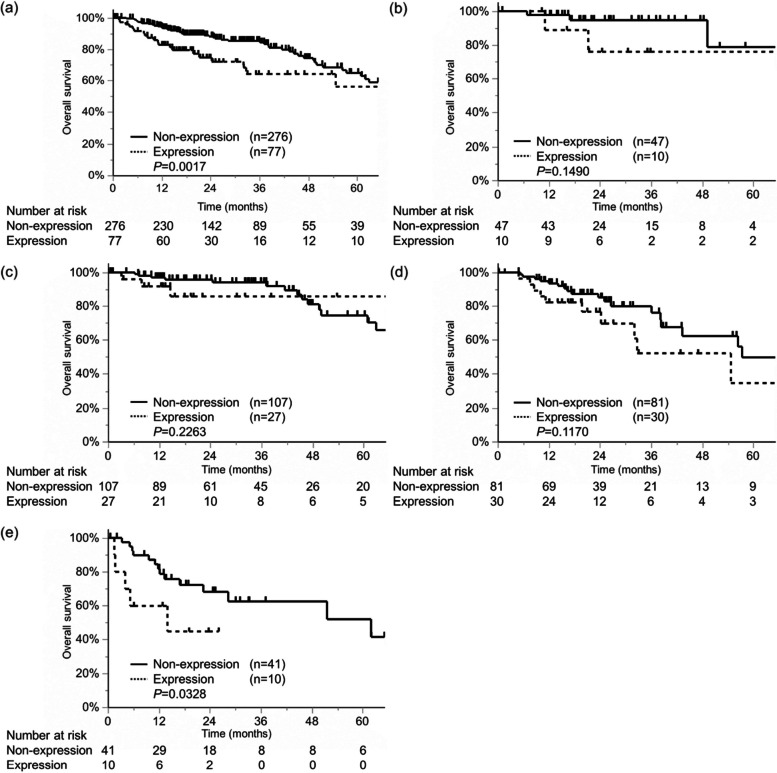
Survival analysis of patients with CRC according to PRTN3-expression from The Cancer Genome Atlas database. The patients are divided into two groups according to PRTN3 expression/non-expression. **a** All patients. **b** Stage I. **c** Stage II. **d** Stage III. **e** Stage IV. Abbreviations: mCRC, metastatic colorectal cancer; PRTN3, proteinase-3

Figure 2 shows the relationship between PRTN3 expression in tumor tissues and CRC prognosis in the TCGA database analysis. Wherein, OS was significantly worse in the expression group than that in the non-expression group (*P*=0.0017) in patients with all stages of CRC (Fig. 2a). While there was no significant difference in OS in Stage I-III, there was a significant difference in OS in Stage IV (*P*=0,0328, Fig. 2b-e). These results suggest that PRTN3 may be associated with the prognosis of mCRC. Therefore, we focused on PRTN3 as a predictive marker for prognosis in mCRC.

### High serum PRTN3 levels: a poor prognostic indicator in mCRC

**Fig. 3:**
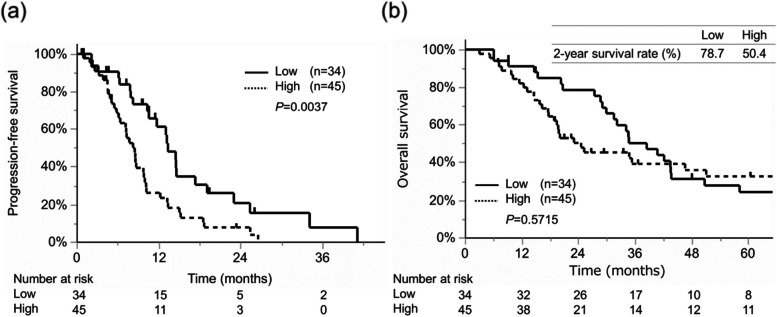
Survival analysis according to serum PRTN3 levels in patients with mCRC. The patients are divided into two groups according to serum PRTN3 levels based on a cutoff value of 21.6 ng/ml identified using time-dependent receiver operating characteristic curve analysis: high expression group (≥21.6 ng/ml) and low expression group (<21.6 ng/ml). **a** Progression-free survival. **b** Overall survival and overall survival rates. Abbreviations: mCRC, metastatic colorectal cancer; PRTN3, proteinase-3

The high (≥21.6 ng/ml) and low (<21.6 ng/ml) PRTN3 expression groups involved 45 and 34 patients, respectively. There were no significant between-group differences in terms of tumor markers, TNM category, histology, or therapeutic interventions including chemotherapy regimen and surgical procedures (Table 1). PRTN3 is a neutrophil-associated protein. However, there was no difference in neutrophil counts between the two groups. Additionally, PFS was significantly worse in the high expression group than in the low expression group (HR, 2.173; 95% CI, 1.269-3.723; *P*=0.037, Fig. 3a). High serum PRTN3 expression (HR, 2.082; 95% CI, 1.188-3.647; *P*=0.010) was an independent risk factor for progression according to the employed multivariate analysis (Table 2).

**Table 1:** Comparison of Clinicopathological Characteristics in mCRC Patients with High and Those with Low PRTN3 Levels

Characteristics	Serum PRTN3 low (n=34)	Serum PRTN3 high (n=45)	*P* -value
Age (years)	67.5 [57.25, 76.25]	66 [55.5, 74.0]	0.593
Sex (male/female)	15/19	26/19	0.229
WBC (/µl)	6395 [4740, 7925]	7290 [5480, 8410]	0.160
Neutrophil count (/µl)	4133 [2803, 5279]	5011 [3593, 6682]	0.089
CEA level (ng/ml)	35.35 [12.95, 90.7]	51.8 [11.4, 331.7]	0.392
CA19-9 level (U/ml)	49.85 [10.6, 485.0]	66.2 [17.9, 260.8]	0.628
Tumor location (right/left)	13/21	21/24	0.454
T category (1/2/3/4)	0/1/11/22	0/1/22/22	0.337
N category (0/1/2/3)	6/15/8/5	11/13/12/9	0.559
Metastasis			
M1 (a/ b/c)	21/5/2	20/10/7	0.181
H (0/1/2/3)	11/11/9/3	14/13/11/7	0.847
PUL (0/1/2/3)	25/5/4/0	30/7/7/1	0.778
P (0/1/2/3)	26/2/3/3	30/3/6/6	0.808
Histological grade (un-/differentiated)	5/29	5/40	0.634
RAS (mutant/wild)	12/22	23/21	0.270
Chemotherapy			
1st line (singlet/doublet/triplet)	7/27/0	9/34/2	0.310
Fluorouracil (+/-)	34/0	45/0	-
Oxaliplatin (+/-)	28/6	34/11	0.467
Irinotecan (+/-)	0/34	4/41	0.074
Anti-VEGF antibody (Bev) (+/-)	10/24	19/26	0.240
Anti-EGFR antibody (+/-)	14/20	11/34	0.114
Number of regimens (1/2/3-)	13/10/11	21/13/11	0.685
Operation			
Primary resection (+/-)	29/5	38/7	0.917
Metastasis resection (+/-)	20/14	24/21	0.626
Radical resection (R0, 1/R2, unresected-)	17/17	19/26	0.492

Data are presented as n or as the median [interquartile range]

*Bev* Bevacizumab, *CA19-9* Carbohydrate antigen19-9, *CEA* Carcinoembryonic antigen, *EGFR* Epidermal growth factor receptor, *H* Hepatic metastasis, *P* Peritoneal metastasis, *PUL* Pulmonary metastasis, *VEGF* Vascular endothelial growth factor, *WBC* White blood cell

**Table 2:** Univariate and multivariate analyses of risk factors of progression-free survival

Factor	Cut-off	Univariate analysis				Multivariate analysis			
		HR	95% CI		*P* -value	HR	95% CI		*P* -value
			Lower	Upper			Lower	Upper	
Serum PRTN3 (ng/ml)	>21.6	2.173	1.269	3.723	0.005	2.082	1.188	3.647	0.010
Age (years)	>65	0.921	0.545	1.555	0.757				
Sex	Male/female	1.162	1.162	1.940	0.565				
WBC count (/µl)	>8600	1.707	0.922	3.162	0.089	1.807	0.964	3.388	0.065
Neutrophil count (/µl)	>4920	1.477	0.884	2.470	0.137				
CEA level (ng/ml)	>6.0	0.893	0.354	2.254	0.812				
CA19-9 level (U/ml)	>37	1.517	0.889	2.588	0.127				
Tumor location	Right/left	1.263	0.756	2.109	0.372				
T category		1.039	0.678	1.638	0.865				
N category		1.360	1.050	1.756	0.020	1.222	0.931	1.604	0.148
Metastasis	a/b/c	1.200	0.808	1.725	0.355				
Operation (radical resection)	R0, 1/R2, unresected	0.619	0.369	1.037	0.068	0.589	0.341	1.018	0.058

Variables selected for the multivariate analysis were those with a univariate analysis *p*-value of < 0.10. *95% CI* 95% confidence interval, *CA19-9* Carbohydrate antigen19-9, *CEA* Carcinoembryonic antigen, *WBC* White blood cell

Although there was no significant between-group difference in OS, the high expression group had a significantly poorer 2-year survival than did the low expression group (50.4% vs. 78.7%; HR, 3.095; 95% CI, 1.331-7.197; *P*=0.009, Fig. 3b). In the multivariate analysis, high serum PRTN3 expression (HR, 4.531; 95% CI, 1.569-13.085; *P*=0.005), high N category (HR, 1.572; 95% CI, 1.056-2.339; *P*=0.026), and radical resection (HR, 0.133; 95% CI, 0.046-0.383; *P*<0.001) were independent risk factors for poor 2-year survival (Table 3). These findings indicated that high serum PRTN3 levels may be associated with poor PFS, which may be related to poor 2-year survival in patients with mCRC.

**Table 3:** Univariate and multivariate analyses of risk factors of 2-year survival

Factor	Cut-off	Univariate analysis				Multivariate analysis			
		HR	95% CI		*P* -value	HR	95% CI		*P* -value
			Lower	Upper			Lower	Upper	
Serum PRTN3 (ng/ml)	>21.6	3.095	1.331	7.197	0.009	4.531	1.569	13.085	0.005
Age (years)	>65	0.786	0.387	1.595	0.505				
Sex	Male/female	1.918	0.919	4.005	0.083	1.890	0.745	4.792	0.180
WBC count (/µl)	>8600	1.018	0.418	2.483	0.968				
Neutrophil count (/µl)	>4920	1.520	0.751	3.075	0.245				
CEA level (ng/ml)	>6.0	4.093	0.557	30.07	0.166				
CA19-9 level (U/ml)	>37	1.146	0.693	3.062	0.321				
Tumor location	Right/left	1.228	0.401	1.653	0.570				
T category		1.001	0.546	1.925	0.997				
N category		1.663	1.160	2.406	0.006	1.572	1.056	2.339	0.026
Metastasis	a/b/c	1.724	1.016	2.814	0.044	1.633	0.903	2.955	0.105
Operation (radical resection)	R0, 1/R2, unresected	0.241	0.103	0.561	0.001	0.133	0.046	0.383	<0.001

Variables selected for the multivariate analysis were those with a univariate analysis *p*-value of < 0.10. *95% CI* 95% confidence interval, *CA19-9 *carbohydrate antigen19-9, *CEA* Carcinoembryonic antigen, *WBC* White blood cell

### Serum PRTN3 levels correlate with stromal PRTN3 expression in neutrophils

**Fig. 4:**
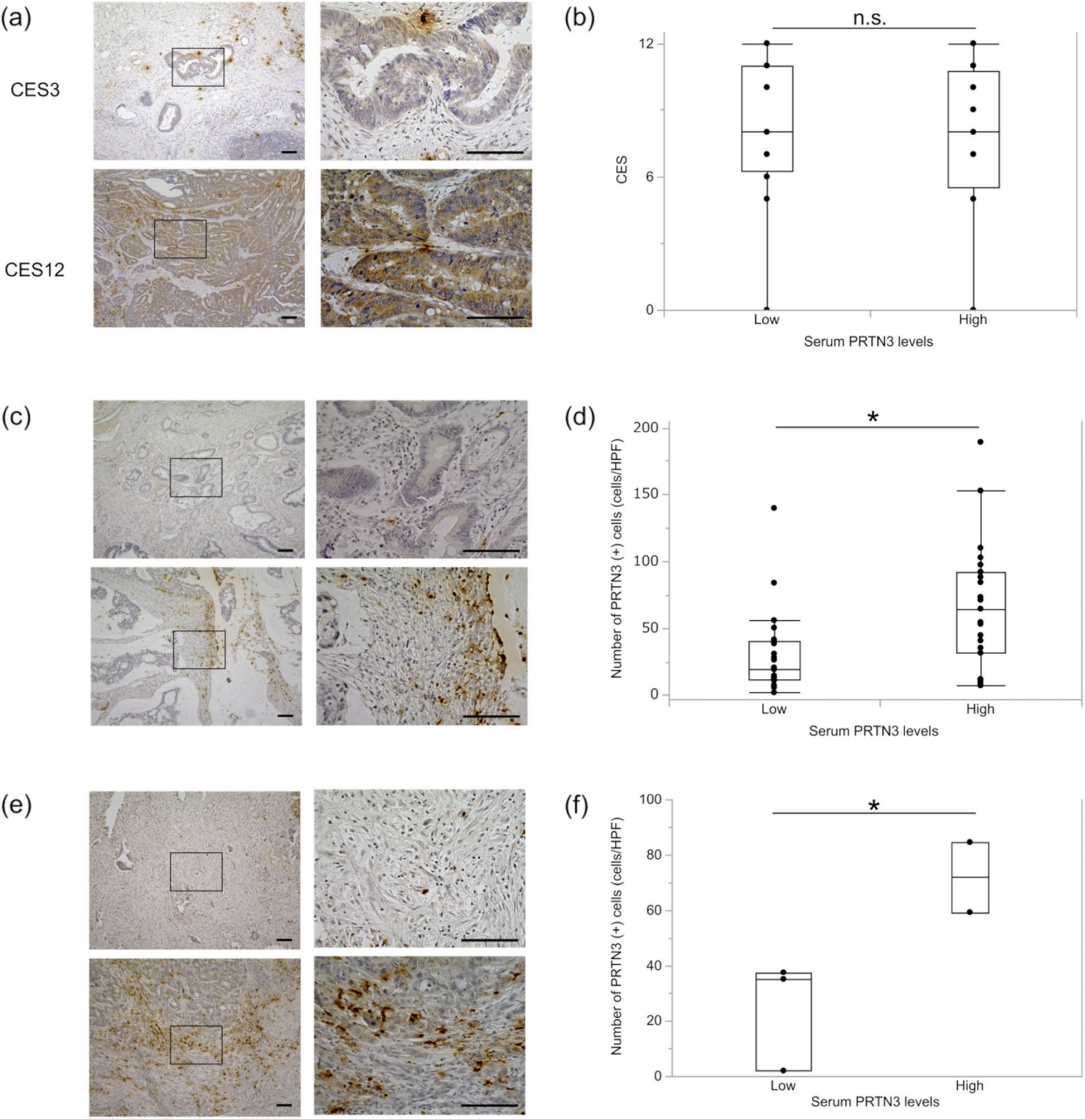
PRTN3 expression in resected specimens. Immunohistochemical (IHC) analysis is performed using primary and metastatic specimens. Scale bar: 100 µm. **a** Representative images of PRTN3 expression in primary tumors. CES=4 × (intensity score − 1) + frequency score. The intensity scores are 0 (negative), 1 (weakly positive), 2 (moderately positive), and 3 (strongly positive). The frequency scores are 1 (5–24%), 2 (25–49%), 3 (50–74%), and 4 (75–100%). **b** Correlation between serum PRTN3 and CES levels. **c** and **e** Representative cases of PRTN3 expression in stromal cells of primary colorectal and metastatic liver specimens. The upper row shows the case with a few positive cells in the stroma. The lower row shows the case with many positive cells in the stroma. **d** and **f** Correlation between serum PRTN3 levels and number of PRTN3-positive cells in the stroma of primary and metastatic tumors, respectively. *P<0.05. Abbreviations: CES, composite expression score; HPF, high-power field; PRTN3, proteinase-3

**Fig. 5:**
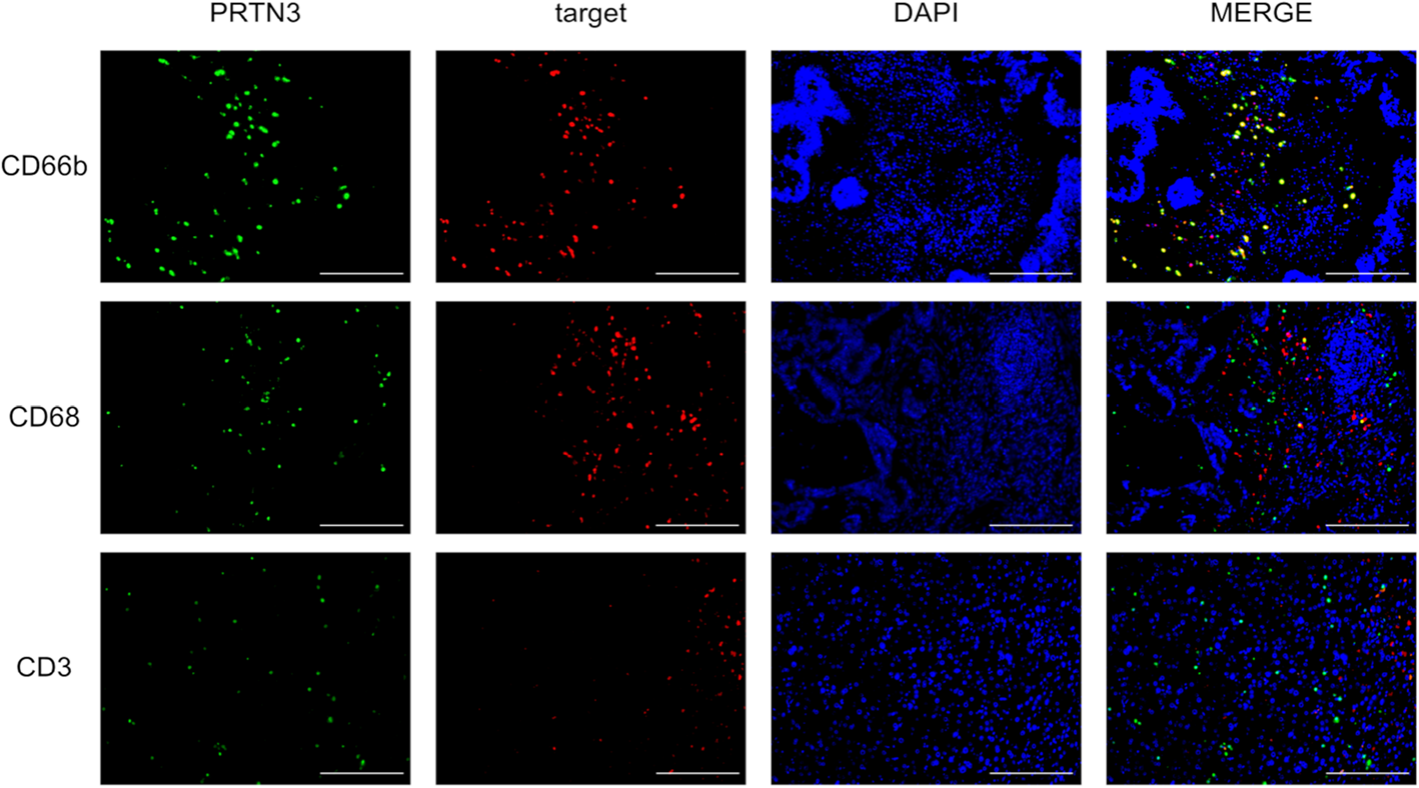
PRTN3 expression in immune cells in the tumor microenvironment. Representative immunofluorescence images of PRTN3 (green), CD66b (red), CD68 (red), CD3 (red), nuclei (blue), and merge (yellow) in the primary tissue. Scale bar: 100 µm. Abbreviations: PRTN3, proteinase-3

In short, IHC analysis (Fig. 4) showed that in the primary tumor, PRTN3 was expressed in both cancer and stromal cells. Serum PRTN3 levels were not correlated with the degree of PRTN3 expression in cancer cells (Fig. 4b). In contrast, the number of PRTN3-positive cells in the stroma was significantly higher in the high serum PRTN3 group than that in the low serum PRTN3 group (*P*=0.0051, Fig. 4c, d). Similar results were observed for liver metastases (*P*=0.035, Fig. 4e, f). In the analysis according to the number of PRTN3-positive cells in the stroma, OS was not significantly different between the high and low groups. However, a high number of PRTN3-positive cells in the stroma were associated with poor PFS (HR, 2.020; 95% CI, 0.962-4.243; *P*=0.063, Supplementary Figure 1). Regarding PRTN3 expression, it was mostly expressed in neutrophils (CD66b) and rarely in lymphocytes (CD3) or macrophages (CD68) (Fig. 5).

### PRTN3 variation in expression from neutrophils has no correlation with its serum levels

**Fig. 6:**
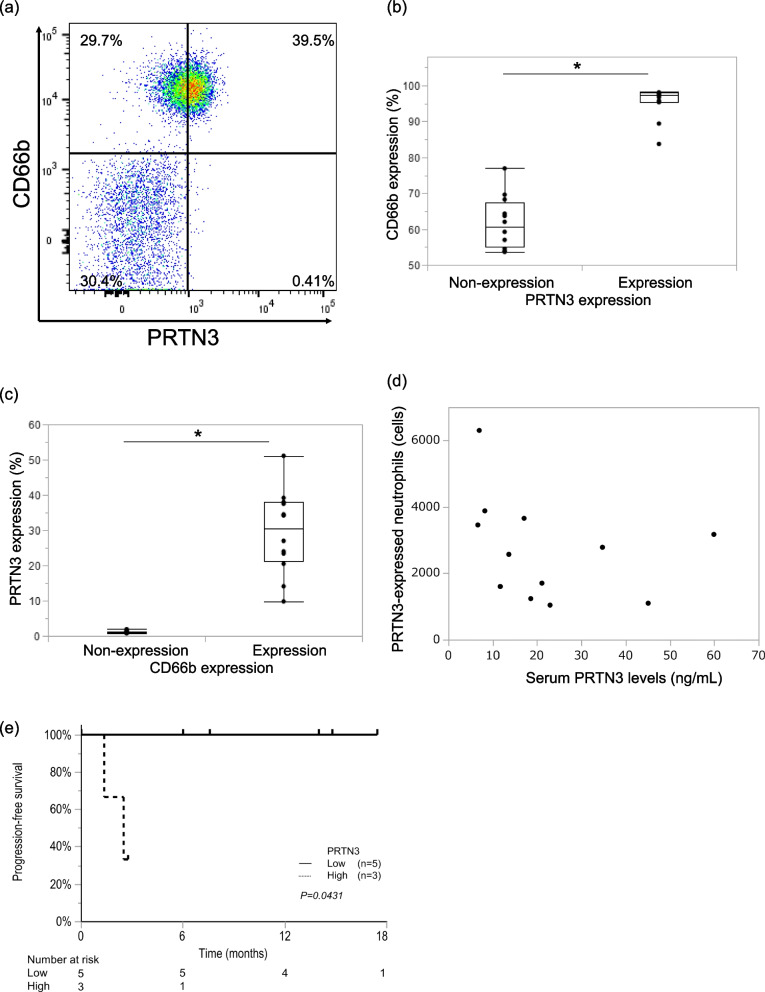
PRTN3 expression in peripheral blood cells of patients with mCRC. **a** Representative data of CD66b/PRTN3 staining of living cells. **b** Percentage of CD66b expression according to PRTN3 expression (n=12). **c** Percentage of PRTN3 expression according to CD66b expression (n=12). (**d**) Correlation between the serum PRTN3 level and the PRTN3-positive neutrophil count (n=12). R=0.3163. P=0.3166. *P<0.05 (e) The relationship between serum PRTN3 levels (the cut-off value is 21.6 ng/ml) and Progression-free survival in the subgroup of 8 patients who received chemotherapy. P=0.043. Abbreviations: mCRC, metastatic colorectal cancer; PRTN3, proteinase-3

The flow cytometric analysis of peripheral blood cells (Fig. 6a) shows representative data of CD66b/PRTN3 staining of living cells and gating. Almost all PRTN3-positive cells expressed CD66b (Fig. 6b). Meanwhile, PRTN3 was expressed in a median of 30.6% (range, 9.81-51.1%) of CD66b-positive cells (Fig. 6c). These results suggest that PRTN3 is expressed in only some neutrophils. However, there was no significant correlation between the number of PRTN3-positive neutrophils and the serum levels of PRTN3 (Fig. 6d). Of the 12 patients included in the flow cytometry analysis, eight received chemotherapy (Supplementary Table 2). Although, this cohort was relatively small, it shows that PFS was significantly poorer in the group with high serum PRTN3 levels than in the group with low levels (*P*=0.043, Fig. 6e).

### Serum PRTN3 levels linked to chemotherapy effectiveness, particularly in bevacizumab subgroup

**Fig. 7:**
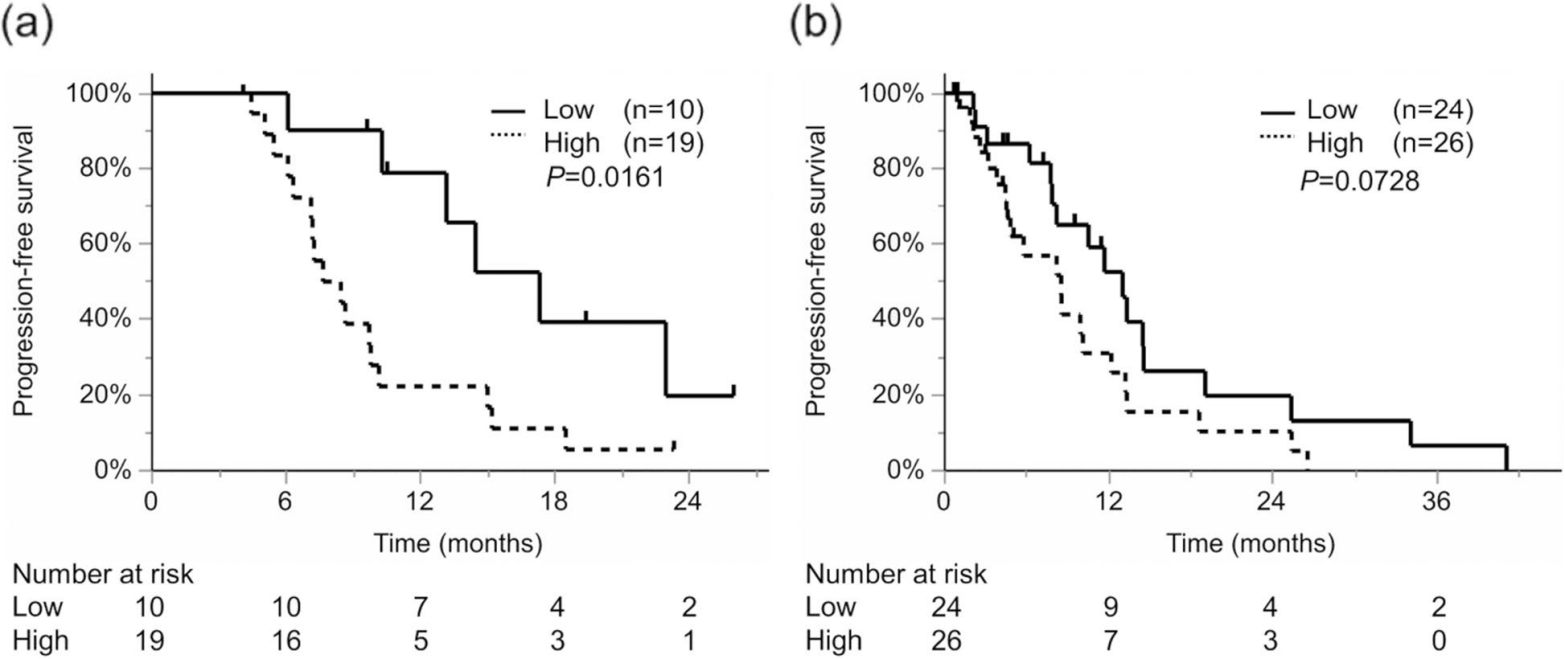
Progression-free survival according to PRTN3 expression in the anti-VEGF antibody treatment subgroups. (a) Progression-free survival of patients treated with bevacizumab. (b) Progression-free survival of patients treated without bevacizumab. *P<0.05

With respect to the association between serum PRTN3 levels and chemosensitivity, the effectiveness of chemotherapy was lower in patients with high serum PRTN3 levels than in those with low serum PRTN3 levels (Table 4). Furthermore, subgroup analyses according to the use of Bev showed that effectiveness of chemotherapy significantly differed regardless of Bev usage (Table 4). However, PFS significantly differed between patients with high and with low PRTN3 expression only in the Bev subgroup (HR, 3.027; 95% CI, 1.175-7.793; *P*=0.0161, Fig. 7). Although more frequent RAS mutations (*P*<0.001) and higher levels of CA19-9 expression (*P*=0.008) were observed in the Bev subgroup, these factors were not significantly associated with PFS in the Bev and no Bev subgroups (*P*=0.8638 and *P*=0.1234) (Supplementary Table 3 and Supplementary Figure 2).

**Table 4:** Best overall response by Response Evaluation Criteria in Solid Tumors

	All specimens			With bevacizumab treatment			Without bevacizumab treatment		
	Serum PRTN3 low (*n*=34)	Serum PRTN3 high (*n*=45)	*P*-value	Serum PRTN3 low (*n*=10)	Serum PRTN3 high (*n*=19)	*P*-value	Serum PRTN3 low (*n*=24)	Serum PRTN3 high (*n*=26)	*P*-value
Best overall response			<0.001			<0.001			0.004
Partial response	21 (61.8)	5 (11.1)		8 (80.0)	2 (10.5)		13 (54.2)	3 (11.5)	
Stable disease	10 (29.4)	27 (60.0)		2 (20.0)	13 (68.4)		8 (33.3)	14 (53.9)	
Progressive disease	3 (8.8)	13 (28.9)		0	4 (21.1)		3 (12.5)	9 (34.6)	

Data are presented as n (%)

*Abbreviations*: *PRTN3* Proteinase-3

### PRTN3 promotes tube formation

**Fig. 8:**
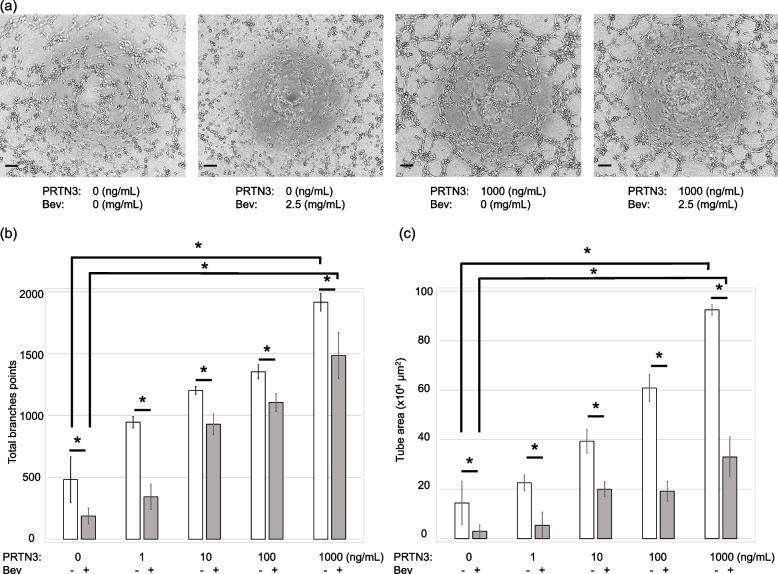
Angiogenic effects of PRTN3 and bevacizumab sensitivity. Tube formation assay was performed using the endothelial tube formation kit. PRTN3 was added at a final concentration of 0-1000 ng/mL, and Bev was added at a final concentration of 0-2.5 mg/mL to 2.5×10^4^ cells of human umbilical vein endothelial cells per each well. After incubation at 37℃ and 5% CO^2^ for 2 h, the number of branches and tube area were evaluated. (a) Representative images of tube formation assay. Scale bar: 100 µm. (b) Number of branching points. The number of branches is increased in a PRTN3 dose-dependent manner. The number of branches is decreased with the administration of bevacizumab. PRTN3 administration increased the number of branches in a dose-dependent manner regardless of bevacizumab administration. (c) Tube area. The results are the same as those for the number of branches. *P<0.05

To support the hypothesis that PRTN3 promotes angiogenesis and that PRTN3 is involved in Bev resistance, a tube formation assay is performed. PRTN3 administration enhanced tube formation in a concentration-dependent manner. On the other hand, while Bev inhibited tube formation, this inhibitory effect was abrogated by the administration of PRTN3 (Fig. 8).

## Discussion

This study is the first to report the importance of serum PRTN3 levels in patients with mCRC. We found that high serum PRTN3 levels were associated with worse PFS (Fig. 3, Table 2) and responses to chemotherapy, especially to Bev chemotherapy, than those associated with low serum PRTN3 levels (Fig. 7, Table 4). Additionally, the tube formation assay confirmed the relationship present between PRTN3 expression and sensitivity to Bev (Fig. 8). These results support that PRTN3 could be a predictive biomarker of PFS by reflecting the response to Bev therapy in patients with mCRC. This could partly be attributed to host factors and the various factors influencing tumor angiogenesis. The TCGA database analysis in the current study showed that the prognosis of only stage IV patients differed according to the status of PRTN3 expression (Fig. 2). Although, detailed information of stage I-III CRC patients couldn’t be obtained from the TCGA database, this result could potentially be attributed to the preferential administration of Bev to stage IV CRC patients. Moreover, this supports our hypothesis that PRTN3 levels are associated with Bev therapy’s response. In our analysis of various cancer types in the TCGA database, we found that the PRTN3-expressing group with gastric cancer had decreased overall survival (Supplementary Figure 3). We hypothesize that this result might also help predict the effect of ramucirumab, an anti-VEGF receptor antibody, which has a similar mechanism. While our study primarily focuses on mCRC, these preliminary findings suggest that the role of PRTN3 in angiogenesis and the chemotherapy response may extent to the patients with stage I-III CRC, other cancer types, and other drugs targeting vascularization. However, additional studies are required to validate this hypothesis.

We found that PRTN3 is expressed in peripheral and stromal neutrophils in patients with mCRC (Figs. 4, 5 and 6). PRTN3 promotes the activation of matrix metalloproteinases (MMP)-2, which are involved in angiogenesis and tumor invasion [21, 22]. Furthermore, PRTN3 is activated by IL-32, which induces protease-activated receptor 2 (PAR2) signaling [33, 34]. One of the downstream pathways of the IL-32-PRTN3-PAR2 axis is the Ras-Raf pathway, which is involved in angiogenesis [33, 35, 36]. The Ras-Raf pathway downstream of VEGF and its receptor signaling is implicated in tumor angiogenesis, and therapies targeting this pathway are commonly used in treating mCRC [5, 37]. Schiffmann et al. reported that a high number of CD177-positive neutrophils in the stroma are associated with poor response to Bev chemotherapy for mCRC, which leads to poor prognosis [38]. These reports support our findings that PRTN3 could be an important regulator of tumor angiogenesis and can thus be a predictive biomarker of Bev response.

Our study showed that serum PRTN3 levels were significantly associated with the number of PRTN3-positive cells in stroma, and almost all PRTN3-positive cells were neutrophils (Figs. 4 and 5). Therefore, we hypothesized that PRTN3 may be play a role in stromal neutrophil function. Considering that tumor-associated neutrophils are involved in tumor angiogenesis [39], the possibility of a relationship existing between neutrophil-associated angiogenesis and Bev sensitivity should be investigated in the future.

Currently, Bev chemotherapy is the standard treatment for mCRC. However, resistance to Bev is a major obstacle to successful treatments [10]. Therefore, predictive biomarkers of Bev response and novel strategies for overcoming resistances to Bev are needed. This study found that PRTN3 may influence the therapeutic effects of Bev (Table 4, Figs. 7 and 8). Serum PRTN3 levels may potentially be beneficial in predicting the efficacy of Bev treatments, and accordingly, facilitate the selection of optimal chemotherapeutic regimen. The PRTN3 level has been reported to promote angiogenesis through MMPs [21, 22], making it a possible independent predictive factor for response, as angiogenesis by PRTN3 may exceed the inhibitory effect of Bev on angiogenesis, resulting in chemotherapy resistance. In addition, from our sub analysis, effectiveness of Bev treatment was not observed in the patients with high PRTN3 levels. Conversely, in the patients with low PRTN3 levels, patients with Bev-treated showed a trend towards improved survival compared to patients without Bev-treated (Supplementary Figure 4). While the small sample size affects the results of our subgroup analysis, these findings suggest the potential of PRTN3 levels to affect decisions regarding Bevacizumab administration. Nevertheless, we emphasize the importance of interpreting these results with caution, as further validation through clinical trials and larger patient cohorts is warranted. Moreover, even if VEGFs are inhibited, the activation of other pathways, stimulated by PRTN3, may result in resistance to chemotherapy. Our tube formation assay results support this hypothesis. PRTN3 is a member of the serine protease family, and its inhibition has shown promise in various contexts, such as in acute lung injuries, and antagonists of PAR2 have been investigated in relation to osteoarthritis [40, 41]. Identification of PRTN3 and its biomarkers might allow optimization of treatment, and PRTN3 antagonists, such as serine protease inhibitors and PAR2 antagonists, may help overcome resistances to Bev. Future research is needed to study these potential therapies.

This study has some limitations, namely, its retrospective, single-center design, and small sample size as well as the advances in chemotherapy during the study period. These limitations may affect the generalizability of our findings. Further studies analyzing the molecular and cellular mechanisms underlying the effects of PRTN3 on tumor angiogenesis using CRC cell lines and neutrophils and the relationship between PRTN3 and stromal neutrophils, are needed to confirm our results.

## Conclusions

In conclusion, serum PRTN3 levels could be a novel predictive biomarker of PFS of first-line chemotherapy in patients with mCRC. In Addition, PRTN3 might predict the efficacy of Bev therapy.

### Supplementary Information

**Additional file 1:** **Supplementary Table 1.** Protein comprehensive proteomics analysis.

**Additional file 2:** **Supplemental Table 2.** Subgroup flow cytometry analysis of treatment response and progression-free survival (*n*=12). Background data of the 12 patients included in the flow cytometry analysis.

**Additional file 3:** **Supplemental Table 3.** Clinicopathological characteristics of mCRC (*n*=79) Bevacizumab background. Comparison of the clinicopathological characteristics of patients with mCRC reciving bevacizumab administration and those not recieving it.

**Additional file 4:** **Supplementary Figure 1.** Progression-free survival according to stromal PRTN3 expression. The patients are divided into two groups according to stromal PRTN3 expression based on a median cutoff value (30 cells / high-power field). *HR*, 2.020; 95% *CI*, 0.962-4.243; *P*=0.063.

**Additional file 5:** **Supplementary Figure 2.** Progression-free survival according to RAS status and serum CA19-9 level. (a) Progression-free survival. The patients are divided into two groups according to RAS status. *P*=0.063. (b) Progression-free survival. The patients are divided into two groups according to serum CA19-9 level based on a cutoff value of 39 U/ml (the standard value of our institution). *P*=0.1234.

**Additional file 6:** **Supplementary Figure 3.** Survival analysis of patients with Gastric cancer according to PRTN3-expression from The Cancer Genome Atlas database. The patients are divided into two groups according to PRTN3 expression/non-expression. (a) All patients. (b) Stage IV. Abbreviations: PRTN3, proteinase-3.

**Additional file 7:** **Supplementary Figure 4.** Progression-free survival of patients with Bevacizumab treatment according to the PRTN3 expression. (a) Progression-free survival of patients treated with bevacizumab in serum PRTN3 low group (b) Progression-free survival of patients treated with bevacizumab in serum PRTN3 high group.

## Data Availability

The datasets used and/or analyzed during the current study are available from the corresponding author on reasonable request.

## Abbreviations

Bev: Bevacizumab
CES: Composite expression score
CRC: Colorectal cancer
ELISA: Enzyme-linked immunosorbent assay
FFPE: Formalin-fixed paraffin-embedded
FPKM: Fragments per kilobase of exon per million
HR: Hazard ratio
IHC: Immunohistochemistry
mAbs: Monoclonal antibodies
mCRC: Metastatic colorectal cancer
MMPs: Matrix metalloproteinases
OS: Overall survival
PAR2: Protease-activated receptor 2
PFS: Progression-free survival
PRTN3: Protenase-3
ROC: Receiver operating characteristic
TCGA: The Cancer Genome Atlas
VEGF: Vascular endothelial growth factor

## References

[1] World Health Organization. Colorectal cancer. https://www.who.int/news-room/fact-sheets/detail/colorectal-cancer. Accessed 13 Dec 2023.

[2] Yoshino T, Arnold D, Taniguchi H, Pentheroudakis G, Yamazaki K, Xu RH (2018). Pan-Asian adapted ESMO consensus guidelines for the management of patients with metastatic colorectal cancer: a JSMO-ESMO initiative endorsed by CSCO, KACO, MOS SSO and TOS. Ann Oncol.

[3] Margonis GA, Vauthey JN (2022). Precision surgery for colorectal liver metastases: Current knowledge and future perspectives. Ann Gastroenterol Surg.

[4] Ogawa H, Yajima T, Sohda M, Shirabe K, Saeki H (2021). Role of surgical resection and its alternative local therapy for pulmonary metastasis of colorectal cancer. Ann Gastroenterol Surg.

[5] Apte RS, Chen DS, Ferrara N (2019). VEGF in signaling and disease: Beyond discovery and development. Cell.

[6] Greiner J, Schmitt M, Li L, Giannopoulos K, Bosch K, Schmitt A (2006). Expression of tumor-associated antigens in acute myeloid leukemia: Implications for specific immunotherapeutic approaches. Blood.

[7] Jain RK (2005). Normalization of tumor vasculature: an emerging concept in antiangiogenic therapy. Science.

[8] Saltz L, Clarke S, Diaz-Rubio E, Scheithauer W, Figer A, Wong R (2023). Bevacizumab in combination with oxaliplatin-based chemotherapy as first-line therapy in metastatic colorectal cancer: a randomized phase III study. J Clin Oncol.

[9] Lin PS, Semrad TJ (2018). Molecular testing for the treatment of advanced colorectal cancer: an overview. Methods Mol Biol.

[10] Huang M, Lin Y, Wang C, Deng L, Chen M, Assaraf YG (2022). New insights into antiangiogenic therapy resistance in cancer: Mechanisms and therapeutic aspects. Drug Resist Updat.

[11] Kitahara M, Hazama S, Tsunedomi R, Takenouchi H, Kanekiyo S, Inoue Y (2016). Prediction of the efficacy of immunotherapy by measuring the integrity of cell-free DNA in plasma in colorectal cancer. Cancer Sci.

[12] Hazama S, Tamada K, Yamaguchi Y, Kawakami Y, Nagano H (2018). Current status of immunotherapy against gastrointestinal cancers and its biomarkers: Perspective for precision immunotherapy. Ann Gastroenterol Surg.

[13] Kono K (2018). Advances in cancer immunotherapy for gastroenterological malignancy. Ann Gastroenterol Surg.

[14] Kijima T, Hazama S, Tsunedomi R, Tanaka H, Takenouchi H, Kanekiyo S (2017). MicroRNA-6826 and −6875 in plasma are valuable non-invasive biomarkers that predict the efficacy of vaccine treatment against metastatic colorectal cancer. Oncol Rep.

[15] Nakashima-Nakasuga C, Hazama S, Suzuki N, Nakagami Y, Xu M, Yoshida S (2020). Serum LOX-1 is a novel prognostic biomarker of colorectal cancer. Int J Clin Oncol.

[16] Yamada K, Hazama S, Suzuki N, Xu M, Nakagami Y, Fujiwara N (2021). Siglec-7 is a predictive biomarker for the efficacy of cancer vaccination against metastatic colorectal cancer. Oncol Lett.

[17] Chidimatsu H, Tsunedomi R, Nakagami Y, Xu M, Nakajima M, Nakashima-Nakasuga C (2023). Serum CCL7 is a novel prognostic biomarker of metastatic colorectal cancer. Anticancer Res.

[18] Campanelli D, Detmers PA, Nathan CF, Gabay JE (1990). Azurocidin and a homologous serine protease from neutrophils. Differential antimicrobial and proteolytic properties. J Clin Invest.

[19] van der Geld YM, Limburg PC, Kallenberg CG (2001). Proteinase 3, Wegener’s autoantigen: from gene to antigen. J Leukoc Biol.

[20] Ge S, Zhu X, Xu Q, Wang J, An C, Hu Y (2022). Neutrophils in ANCA-associated vasculitis: Mechanisms and implications for management. Front Pharmacol.

[21] Shamamian P, Pocock BJ, Schwartz JD, Monea S, Chuang N, Whiting D (2000). Neutrophil-derived serine proteinases enhance membrane type-1 matrix metalloproteinase-dependent tumor cell invasion. Surgery.

[22] Shamamian P, Schwartz JD, Pocock BJ, Monea S, Whiting D, Marcus SG (2001). Activation of progelatinase A (MMP-2) by neutrophil elastase, cathepsin G, and proteinase-3: a role for inflammatory cells in tumor invasion and angiogenesis. J Cell Physiol.

[23] Hu D, Ansari D, Zhou Q, Sasor A, Said Hilmersson K, Andersson R (2019). Low P4HA2 and high PRTN3 expression predicts poor survival in patients with pancreatic cancer. Scand J Gastroenterol.

[24] Wei Z, Wu B, Wang L, Zhang J (2020). A large-scale transcriptome analysis identified ELANE and PRTN3 as novel methylation prognostic signatures for clear cell renal cell carcinoma. J Cell Physiol.

[25] Fatalska A, Rusetska N, Bakuła-Zalewska E, Kowalik A, Zięba S, Wroblewska A et al. Inflammatory proteins HMGA2 and PRTN3 as drivers of vulvar squamous cell carcinoma progression. Cancers (Basel) 2020;13. 10.3390/cancers13010027 10.3390/cancers13010027 PMC7793473 33374674

[26] The Cancer Genome Atlas database. PanCancer Atlas. https://portal.gdc.cancer.gov/ Accessed 13 Dec 2023.

[27] Wang J, Wang X, Guo Y, Ye L, Li D, Hu A (2021). Therapeutic targeting of SPIB/SPI1-facilitated interplay of cancer cells and neutrophils inhibits aerobic glycolysis and cancer progression. Clin Transl Med.

[28] Stryker Z, Rajabi M, Davis P, Mousa SA (2019). Evaluation of angiogenesis assays. Biomedicines.

[29] Weng M, Yue Y, Wu D, Zhou C, Guo M, Sun C (2022). Increased MPO in colorectal cancer is associated with high peripheral neutrophil counts and a poor prognosis: A TCGA with propensity score-matched analysis. Front Oncol.

[30] Kemik O, Sumer A, Kemik AS, Hasirci I, Purisa S, Dulger AC (2010). The relationship among acute-phase response proteins, cytokines and hormones in cachectic patients with colon cancer. World J Surg Oncol.

[31] Zhou JM, Jiang H, Yuan T, Zhou GX, Li XB, Wen KM (2019). High hnRNP AB expression is associated with poor prognosis in patients with colorectal cancer. Oncol Lett.

[32] Ishigami SI, Arii S, Furutani M, Niwano M, Harada T, Mizumoto M (1998). Predictive value of vascular endothelial growth factor (VEGF) in metastasis and prognosis of human colorectal cancer. Br J Cancer.

[33] Nakayama M, Niki Y, Kawasaki T, Takeda Y, Ikegami H, Toyama Y (2013). IL-32-PAR2 axis is an innate immunity sensor providing alternative signaling for LPS-TRIF axis. Sci Rep.

[34] Novick D, Rubinstein M, Azam T, Rabinkov A, Dinarello CA, Kim SH (2006). Proteinase 3 is an IL-32 binding protein. Proc Natl Acad Sci U S A.

[35] Kawaguchi M, Yamamoto K, Kataoka H, Izumi A, Yamashita F, Kiwaki T, Nishida T, Camerer E, Fukushima T (2020). Protease-activated receptor-2 accelerates intestinal tumor formation through activation of nuclear factor-κB signaling and tumor angiogenesis in ApcMin/+ mice. Cancer Sci.

[36] Pawar NR, Buzza MS, Antalis TM (2019). Membrane-anchored serine proteases and protease-activated Receptor-2-Mediated signaling: Co-conspirators in cancer progression. Cancer Res.

[37] Ferrara N, Adamis AP (2016). Ten years of anti-vascular endothelial growth factor therapy. Nat Rev Drug Discov.

[38] Schiffmann LM, Fritsch M, Gebauer F, Günther SD, Stair NR, Seeger JM, Thangarajah F, Dieplinger G, Bludau M, Alakus H, Göbel H (2019). Tumour-infiltrating neutrophils counteract anti-VEGF therapy in metastatic colorectal cancer. Br J Cancer.

[39] Jaillon S, Ponzetta A, Di Mitri D, Santoni A, Bonecchi R, Mantovani A (2020). Neutrophil diversity and plasticity in tumour progression and therapy. Nat Rev Cancer.

[40] Hwang TL, Wang WH, Wang TY, Yu HP, Hsieh PW (2015). Synthesis and pharmacological characterization of 2-aminobenzaldehyde oxime analogs as dual inhibitors of neutrophil elastase and proteinase 3. Bioorg Med Chem.

[41] Huang X, Ni B, Xi Y, Chu X, Zhang R, You H (2019). Protease-activated receptor 2 (PAR-2) antagonist AZ3451 as a novel therapeutic agent for osteoarthritis. Aging (Albany NY).

