# Supplementary material for: High serum proteinase-3 levels predict poor progression-free survival and lower efficacy of bevacizumab in metastatic colorectal cancer
Source: BMC Cancer. 2024 Feb 2;24:165. doi: 10.1186/s12885-024-11924-4 (PMC10835931; doi:10.1186/s12885-024-11924-4)
Supplement: Supplementary file 1 — Additional file 1: Supplementary Table 1. Protein comprehensive proteomics analysis. [file 12885_2024_11924_MOESM1_ESM.docx]

**Supplemental Table 1. Protein comprehensive proteomics analysis**

| Serum sample | | | | | |  | Tissue sample | | | | | |
| --- | --- | --- | --- | --- | --- | --- | --- | --- | --- | --- | --- | --- |
| Rank | Proteins | OS ≥3  (n=9)  Mean | OS<2  (n=11)  Mean | Fisher’s ratio | *P*-value |  | Rank | Proteins | OS ≥3 (n=9)  Mean | OS<2  (n=11)  Mean | Fisher’s ratio | *P* -value |
| 1 | MPO | 29407.4 | 51014.8 | 4.65 | <0.001 |  | 1 | B7-H2 | 16059.9 | 51353.8 | 2.25 | 0.050 |
| 2 | PRTN3 | 7979.2 | 15841.6 | 3.44 | <0.001 |  | 2 | RETN | 1154.2 | 2410.2 | 1.91 | 0.045 |
| 3 | OLR1 | 460.7 | 752.5 | 2.74 | 0.004 |  | 3 | LYZ | 9132.7 | 21434.3 | 1.74 | 0.063 |
| 4 | IL-8 | 3318.9 | 7096.9 | 2.59 | 0.008 |  | 4 | MPO | 8607.6 | 32262.0 | 1.60 | 0.078 |
| 5 | LTF | 8649.9 | 14674.9 | 2.26 | 0.003 |  | 5 | PRTN3 | 10824.1 | 37279.8 | 1.50 | 0.095 |
| 6 | BPI | 3032.8 | 5904.1 | 2.03 | 0.011 |  | 6 | LTF | 33540.5 | 103092.6 | 1.43 | 0.085 |
| 7 | hnRNP A2/B1 | 4424.2 | 7377.9 | 1.94 | 0.018 |  | 7 | FGR | 201.4 | 451.5 | 1.38 | 0.080 |
| 8 | PGLYRP1 | 948.4 | 1503.8 | 1.79 | 0.008 |  | 8 | HIST1H1C | 2266.3 | 6839.2 | 1.38 | 0.037 |
| 9 | VEGF121 | 4635.7 | 11089.3 | 1.55 | 0.021 |  | 9 | ALP | 56779.7 | 133198.1 | 1.33 | 0.072 |
| 10 | CCL7 | 2773.6 | 5455.0 | 1.21 | 0.107 |  | 10 | TGM3 | 2036.7 | 166.0 | 1.32 | 0.234 |

ALP, alkaline phosphatase, tissue-nonspecific isozyme; B7-H2, ICOS ligand; BPI, bactericidal permeability-increasing protein; CCL7, C-C motif chemokine 7; FGR, tyrosine-protein kinase Fgr; HIST1H1C, histone cluster 1, H1c; hnRNP A2/B1, heterogeneous nuclear ribonucleoproteins A2/B1; IL-8, interleukin-8; LTF, lactoferrin; LYZ, lysozyme; MPO, myeloperoxidase; OLR1, oxidized low-density lipoprotein receptor 1; PGLYRP1, peptidoglycan recognition protein 1; PRTN3, proteinase-3; RETN, resistin; TGM3, protein-glutamine gamma-glutamyltransferase E; VEGF121, vascular endothelial growth factor A, isoform 121
