# Supplementary material for: High serum proteinase-3 levels predict poor progression-free survival and lower efficacy of bevacizumab in metastatic colorectal cancer
Source: BMC Cancer. 2024 Feb 2;24:165. doi: 10.1186/s12885-024-11924-4 (PMC10835931; doi:10.1186/s12885-024-11924-4)
Supplement: Supplementary file 2 — Additional file 2: Supplemental Table 2. Subgroup flow cytometry analysis of treatment response and progression-free survival (n=12). Background data of the 12 patients included in the flow cytometry analysis. [file 12885_2024_11924_MOESM2_ESM.docx]

**Supplemental Table 2. Background data of the 12 patients included in the flow cytometry analysis**

| Case | PRTN3 levels (ng/ml) | CD66+PRTN3+ (%) | Chemotherapy | PFS (M) |
| --- | --- | --- | --- | --- |
| 1 | 6.7 | 24 | no | - |
| 2 | 7.0 | 34.2 | no | - |
| 3 | 8.3 | 39.2 | yes | 14.0 |
| 4 | 11.7 | 38 | yes | 14.8 |
| 5 | 13.6 | 34.5 | yes | 7.6 |
| 6 | 17.6 | 37.5 | yes | 17.4 |
| 7 | 18.6 | 51.1 | no | - |
| 8 | 21.1 | 23.4 | yes | 6.0 |
| 9 | 22.9 | 9.81 | yes | 2.8 |
| 10 | 34.8 | 27 | yes | 1.3 |
| 11 | 45.1 | 14.1 | yes | 2.5 |
| 12 | 59.9 | 20.5 | no | - |

PFS, progression-free survival; PRTN3, proteinase-3
