# Supplementary material for: High serum proteinase-3 levels predict poor progression-free survival and lower efficacy of bevacizumab in metastatic colorectal cancer
Source: BMC Cancer. 2024 Feb 2;24:165. doi: 10.1186/s12885-024-11924-4 (PMC10835931; doi:10.1186/s12885-024-11924-4)
Supplement: Supplementary file 3 — Additional file 3: Supplemental Table 3. Clinicopathological characteristics of mCRC (n=79) Bevacizumab background. Comparison of the clinicopathological characteristics of patients with mCRC reciving bevacizumab administration and those not recieving it. [file 12885_2024_11924_MOESM3_ESM.docx]

**Supplemental Table 3. Comparison of the clinicopathological characteristics of patients with mCRC reciving bevacizumab administration and those not recieving it**

| Characteristics | With Bev  (n=29) | Without Bev  (n=50) | *P* -value |
| --- | --- | --- | --- |
| PRTN3 (high/low) | 19/10 | 26/24 | 0.240 |
| Age (years) | 69 [62.0, 77.0] | 65 [55.0, 73.5] | 0.165 |
| Sex (Male/Female) | 10/19 | 31/19 | 0.018 |
| WBC (/µl) | 6200 [5020, 8285] | 6700 [5450, 8465] | 0.726 |
| Neutrophils | 4345 [3025, 6625] | 4732 [3613, 6088] | 0.555 |
| CEA (ng/ml) | 78.2 [15.2, 223.1] | 37.3 [10.2, 122.9] | 0.191 |
| CA19-9 (U/ml) | 106.4 [42.9, 993.2] | 31.7 [10.7, 129.7] | 0.008 |
| Tumor location (Right/Left) | 16/13 | 18/32 | 0.097 |
| T category (1/2/3/4) | 0/0/10/19 | 0/2/23/25 | 0.201 |
| N category (0/1/2/3) | 6/10/7/6 | 11/18/13/8 | 0.965 |
| Metastasis |  |  |  |
| M1(a/ｂ/c) | 13/7/2 | 28/8/7 | 0.424 |
| H (0/1/2/3) | 9/6/9/5 | 16/18/11/5 | 0.421 |
| PUL (0/1/2/3) | 18/3/7/1 | 37/9/4/0 | 0.094 |
| P (0/1/2/3) | 20/2/2/5 | 36/3/7/4 | 0.525 |
| Histological grade (un-/differentiated) | 2/27 | 8/42 | 0.222 |
| RAS (mutant/wild) | 21/8 | 12/37 | <0.001 |
| Chemotherapy |  |  |  |
| 1st line (singlet/doublet/triplet) |  |  |  |
| Fluorouracil (+/-) | 29/0 | 50/0 | - |
| Oxaliplatin (+/-) | 28/1 | 34/16 | 0.001 |
| Irinotecan (+/-) | 3/26 | 1/49 | 0.109 |
| Anti-VEGF antibody (+/-) | 29/0 | 0/50 | <0.001 |
| Anti-EGFR antibody (+/-) | 0/29 | 25/25 | <0.001 |
| Number of regimens (1/2/3-) | 7/11/11 | 27/12/11 | 0.031 |
| Operation |  |  |  |
| Primary resection (+/-) | 24/5 | 43/7 | 0.700 |
| Metastasis resection (+/-) | 16/13 | 28/22 | 0.943 |
| Radical resection (+/-) | 12/17 | 24/26 | 0.568 |

Data are presented as n or as the median [interquartile range].

Bev, bevacizumab; CA19-9, carbohydrate antigen19-9; CEA, carcinoembryonic antigen; EGFR, Epidermal growth factor receptor; H, hepatic metastasis; P, peritoneal metastasis; PUL, pulmonary metastasis; VEGF, vascular endothelial growth factor; WBC, white blood cell
