# Supplementary material for: High serum proteinase-3 levels predict poor progression-free survival and lower efficacy of bevacizumab in metastatic colorectal cancer
Source: BMC Cancer. 2024 Feb 2;24:165. doi: 10.1186/s12885-024-11924-4 (PMC10835931; doi:10.1186/s12885-024-11924-4)
Supplement: Supplementary file 4 — Additional file 4: Supplementary Figure 1. Progression-free survival according to stromal PRTN3 expression. The patients are divided into two groups according to stromal PRTN3 expression based on a median cutoff value (30 cells / high-power field). HR, 2.020; 95% CI, 0.962-4.243; P=0.063. [file 12885_2024_11924_MOESM4_ESM.pdf]

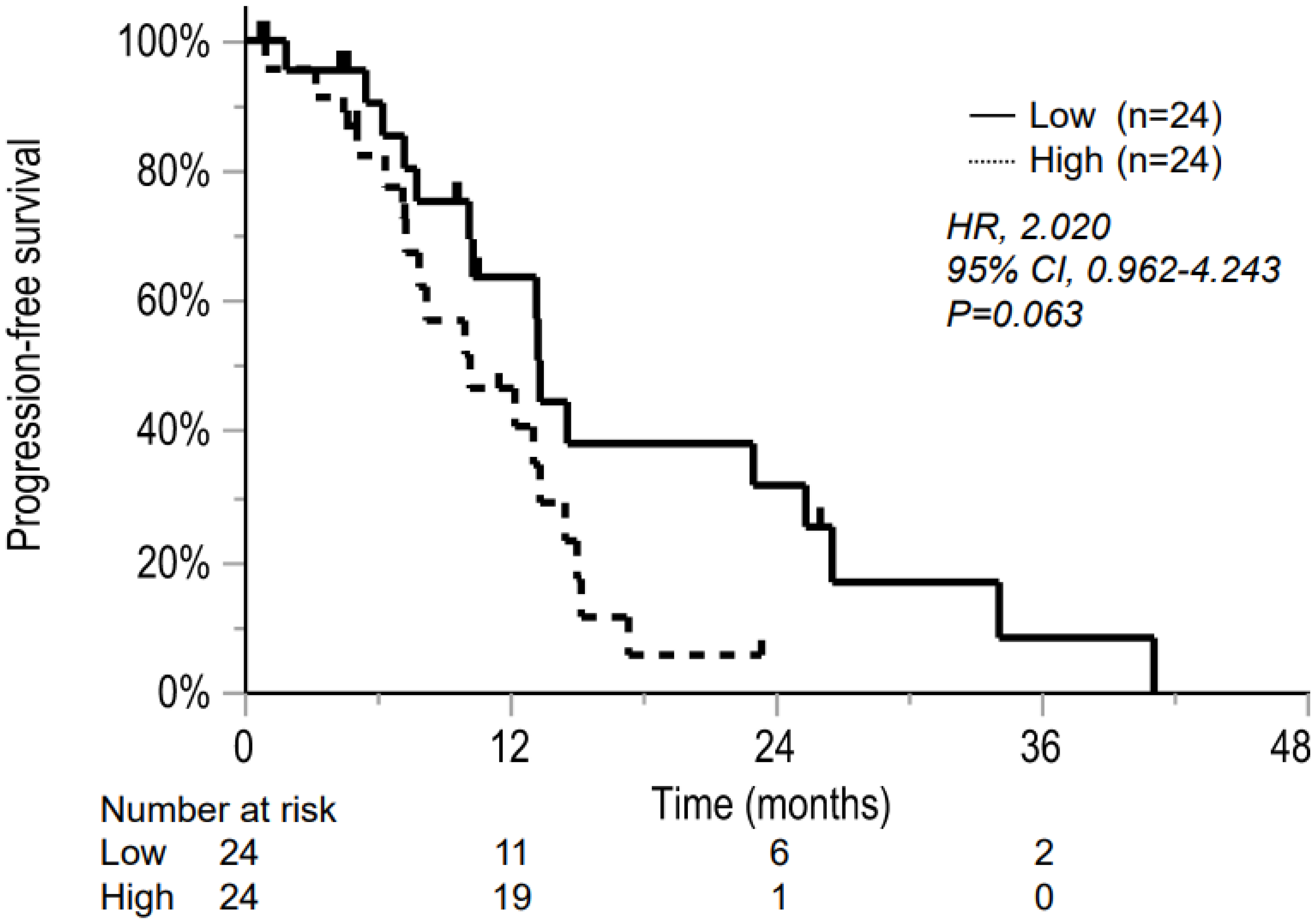

Supplementary Figure 1. Progression-free survival according to stromal PRTN3 expression
The patients are divided into two groups according to stromal PRTN3 expression based on a median cutoff value (30 cells / high-power field). HR, 2.020; 95% CI, 0.962-4.243; P=0.063.
