# Supplementary material for: High serum proteinase-3 levels predict poor progression-free survival and lower efficacy of bevacizumab in metastatic colorectal cancer
Source: BMC Cancer. 2024 Feb 2;24:165. doi: 10.1186/s12885-024-11924-4 (PMC10835931; doi:10.1186/s12885-024-11924-4)
Supplement: Supplementary file 5 — Additional file 5: Supplementary Figure 2. Progression-free survival according to RAS status and serum CA19-9 level. (a) Progression-free survival. The patients are divided into two groups according to RAS status. P=0.063. (b) Progression-free survival. The patients are divided into two groups according to serum CA19-9 level based on a cutoff value of 39 U/ml (the standard value of our institution). P=0.1234. [file 12885_2024_11924_MOESM5_ESM.pdf]

(a)

(b)

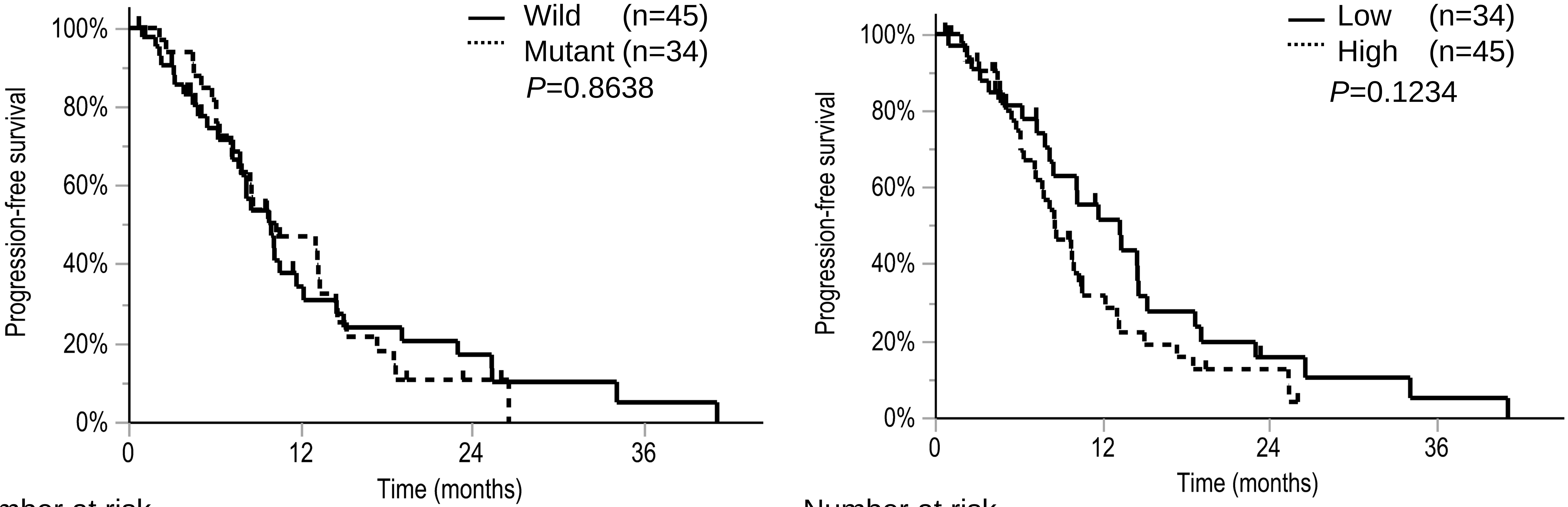

Number at risk				
Wild	45	11	6	2
High	34	14	2	1

Number at risk				
Low	34	14	4	2
High	45	12	4	1

Supplementary Figure 2. Progression-free survival according to RAS status and serum CA19-9 level
(a) Progression-free survival. The patients are divided into two groups according to RAS status. P=0.063.
(b) Progression-free survival. The patients are divided into two groups according to serum CA19-9 level based on a cutoff value of 39 U/ml (the standard value of our institution). P=0.1234.
