# Supplementary material for: High serum proteinase-3 levels predict poor progression-free survival and lower efficacy of bevacizumab in metastatic colorectal cancer
Source: BMC Cancer. 2024 Feb 2;24:165. doi: 10.1186/s12885-024-11924-4 (PMC10835931; doi:10.1186/s12885-024-11924-4)
Supplement: Supplementary file 6 — Additional file 6: Supplementary Figure 3. Survival analysis of patients with Gastric cancer according to PRTN3-expression from The Cancer Genome Atlas database. The patients are divided into two groups according to PRTN3 expression/non-expression. (a) All patients. (b) Stage IV. Abbreviations: PRTN3, proteinase-3. [file 12885_2024_11924_MOESM6_ESM.pdf]

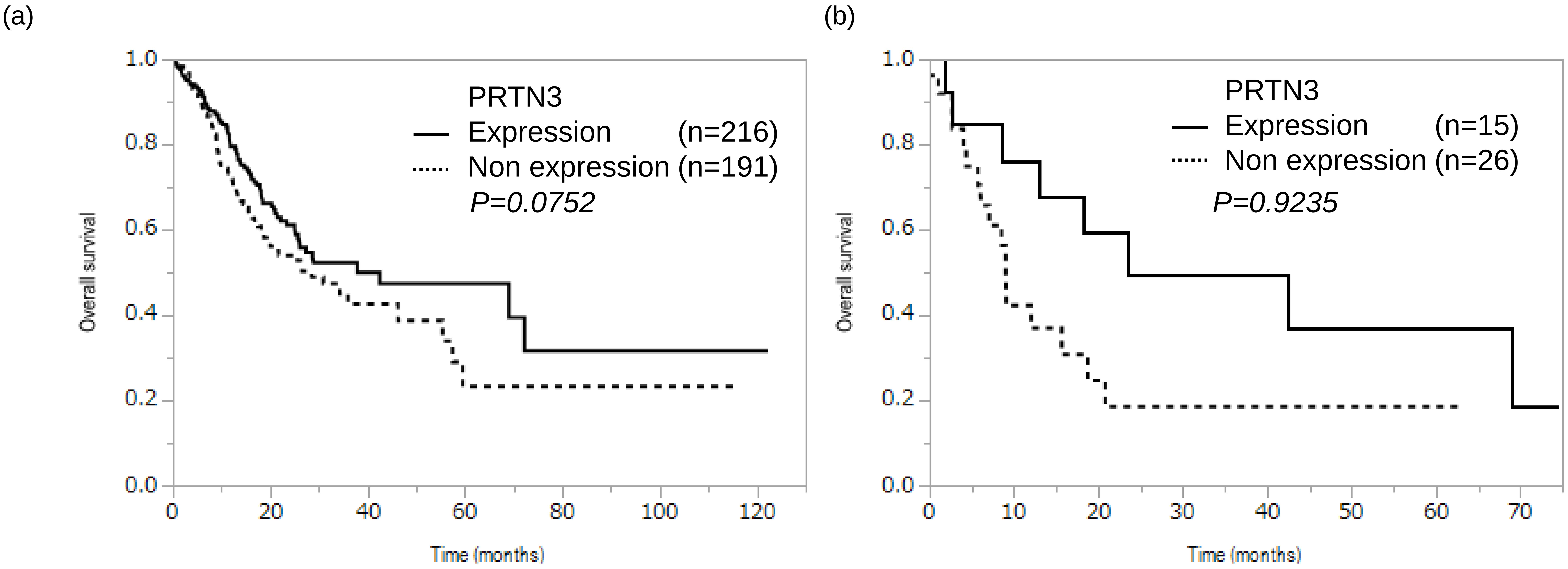

Supplementary Figure 3. Survival analysis of patients with Gastric cancer according to PRTN3-expression from The Cancer Genome Atlas database

The patients are divided into two groups according to PRTN3 expression/non-expression.

(a) All patients. (b) Stage IV

Abbreviations: PRTN3, proteinase-3
