# Supplementary material for: High serum proteinase-3 levels predict poor progression-free survival and lower efficacy of bevacizumab in metastatic colorectal cancer
Source: BMC Cancer. 2024 Feb 2;24:165. doi: 10.1186/s12885-024-11924-4 (PMC10835931; doi:10.1186/s12885-024-11924-4)
Supplement: Supplementary file 7 — Additional file 7: Supplementary Figure 4. Progression-free survival of patients with Bevacizumab treatment according to the PRTN3 expression. (a) Progression-free survival of patients treated with bevacizumab in serum PRTN3 low group (b) Progression-free survival of patients treated with bevacizumab in serum PRTN3 high group. [file 12885_2024_11924_MOESM7_ESM.pdf]

(a) (b)

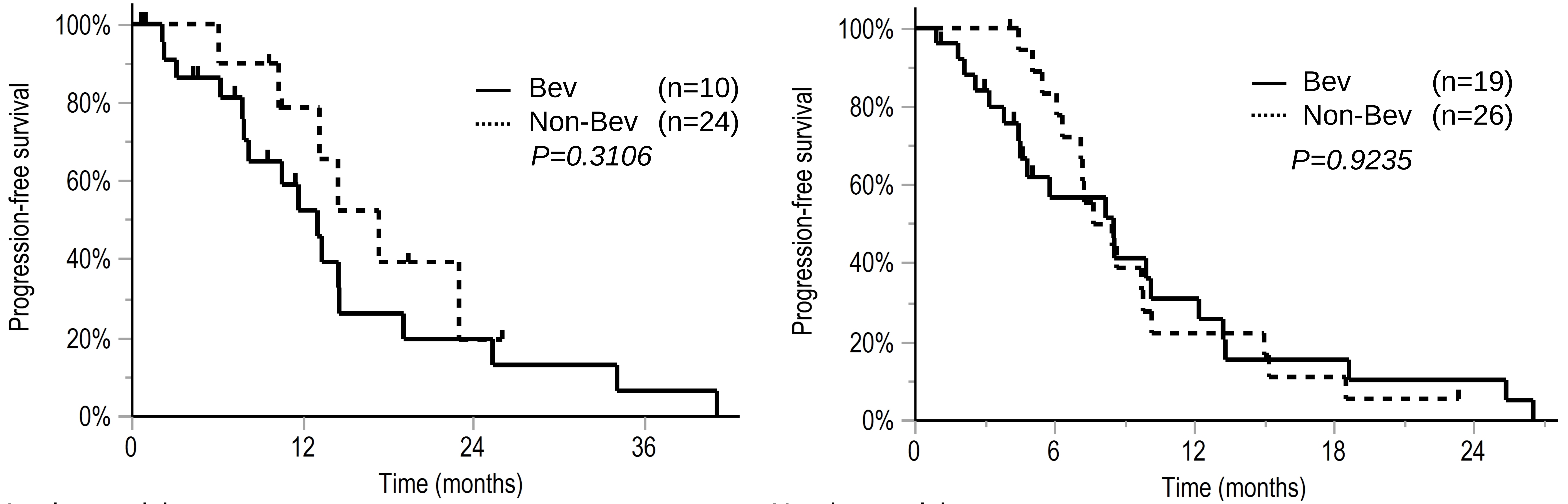

(a) Number at risk

Bev	10	7	2	0
Non-Bev	24	9	4	2

(b) Number at risk

Bev	19	16	5	3	1
Non-Bev	26	12	7	4	3

Supplementary Figure 4. Progression-free survival of patients with Bevacizumab treatment according to the PRTN3 expression

(a) Progression-free survival of patients treated with bevacizumab in serum PRTN3 low group (b) Progression-free survival of patients treated with bevacizumab in serum PRTN3 high group.
